# Harnessing the Farm Effect: Microbial Products for the Treatment and Prevention of Asthma Throughout Life

**DOI:** 10.1111/imr.70012

**Published:** 2025-03-04

**Authors:** Maile K. Hollinger, Emily M. Grayson, Caroline M. Ferreira, Anne I. Sperling

**Affiliations:** ^1^ Beirne B. Carter Center for Immunology Research University of Virginia Charlottesville Virginia USA; ^2^ Department of Medicine, Pulmonary and Critical Care University of Virginia Charlottesville Virginia USA; ^3^ Department of Microbiology, Immunology, and Cancer Biology University of Virginia Charlottesville Virginia USA; ^4^ Institute of Environmental, Chemistry and Pharmaceutics Sciences, Department of Pharmaceutics Sciences Federal University of São Paulo São Paulo Brazil

**Keywords:** asthma, farm effect, microbiota, postbiotic, prebiotic, probiotic, short chain fatty acid

## Abstract

It has long been appreciated that farm exposure early in life protects individuals from allergic asthma. Understanding what component(s) of this exposure is responsible for this protection is crucial to understanding allergic asthma pathogenesis and developing strategies to prevent or treat allergic asthma. In this review, we introduce the concept of Farm‐Friends, or specific microbes associated with both a farm environment and protection from allergic asthma. We review the mechanism(s) by which these Farm‐Friends suppress allergic inflammation, with a focus on the molecule(s) produced by these Farm‐Friends. Finally, we discuss the relevance of Farm‐Friend administration (oral vs. inhaled) for preventing the development and severity of allergic asthma throughout childhood and adulthood. By developing a fuller understanding of which Farm‐Friends modulate host immunity, a greater wealth of prophylactic and therapeutic options becomes available to counter the current allergy epidemic.

## 1 Introduction

Asthma is a chronic inflammatory disease of the airways, influenced by environmental and genetic factors that affect its development and severity. Asthma has several overlapping phenotypes with specific clinical and physiological characteristics, but in this review, we will focus on allergic asthma, which is the most common endotype. Allergic asthma affects 1 in 13 people in the U.S. alone and results in over 1000 deaths per day worldwide [1]. Asthma prevalence can be further stratified by sex and age, as women are much more likely to have asthma with greater severity than male counterparts after puberty [2]. Intriguingly, clinical asthma prevalence has steadily increased [3], and the reason for this has been the subject of extensive investigation. Understanding environmental and genetic factors of asthma risk and severity, and how they interact to cause disease, is of utmost importance for the prevention and development of treatments.

Asthma risk is widely heterogeneous, but can be associated with age, sex, genetics, and the microbiome. A family history of asthma, or other allergic or atopic diseases, is a common risk factor for a child to develop asthma, and this seems to be heightened if the disease is afflicting the mother as opposed to the father. One study correlating maternal total IgE levels with offspring atopic disease was found to have up to 83% specificity at predicting child atopy [4]. Furthermore, genome‐wide association studies have evaluated genes and single nucleotide polymorphisms (SNPs) that correlate with asthma risk. Hundreds of genes have been determined to be associated with asthma or atopy, and the genes that impact childhood‐onset and adult‐onset asthma are not completely overlapping [5]. These factors can contribute not only to the onset of disease, but are also believed to play a role in asthma severity [6], and the interplay between these genes and the environment to facilitate the asthma phenotype remains an active area of research [7].

Over the past 60 years, there has been a temporal trend of increasing asthma prevalence in first‐world countries, in large part due to the urbanization of infrastructure. A more urban lifestyle involving less allergenic exposure has been increasingly associated with asthma. In 1989, a study of more than 17,000 British children observed that babies born in a household with many siblings were less susceptible to eczema in the first year of life and hay fever later on [8]. This suggested that the transmission of some contagious diseases from older siblings to younger ones could protect against allergies, leading to the formation of the so‐called hygiene hypothesis.

One of the immunological explanations for this protection was that infections increase the Th1 immune response and simultaneously decrease the Th2 response, and therefore an increase in IFNγ would inhibit the triggering of Th2 inflammation [9]. However, this hypothesis has been the subject of scrutiny, as researchers in Africa observed that children on that continent had increased Th2 inflammation but were also protected from allergies [10, 11]. While seemingly paradoxical, the increase in Th2 responses corresponded to increased IL‐10, which was inhibiting allergic inflammation [10, 11, 12]. These findings, combined with the discovery of regulatory T cells (Tregs) and their influence on asthma just before the turn of the 21st century [13, 14, 15], led to the term hygiene hypothesis being modified to the Old Friends Hypothesis, as it was understood that the protection was not specifically related to hygiene [16].

In the Old Friends Hypothesis, it is posited that the tolerogenic regulation related to allergy and asthma protection involves not only infections but also the colonization of our body, especially the gut, by microorganisms [17]. Some microorganisms carve out a niche in the body and provide benefits to the host, thereby becoming commensals. Other microorganisms, such as those colonizing soil or animals, pass transiently through human hosts but are sensed by the host immune system [17]. A corollary of the Old Friends hypothesis, the Farm Effect, focuses on the microbial exposures lost as modern society became more divorced from food production. Developed after the discovery that children raised on farms were less susceptible to asthma and allergy, the Farm Effect argues that colonization by and exposure to microorganisms associated with agriculture, animal rearing, and consumption of less‐processed foods “trains” the immune system to become hyporesponsive to allergens later in life. Therefore, during this early‐life “window of opportunity,” these microbial exposures, as well as other important factors such as the use of antibiotics and medications, type of food, and lifestyle, are paramount to prevent allergic sensitization [18, 19, 20, 21, 22, 23].

**FIGURE 1:**
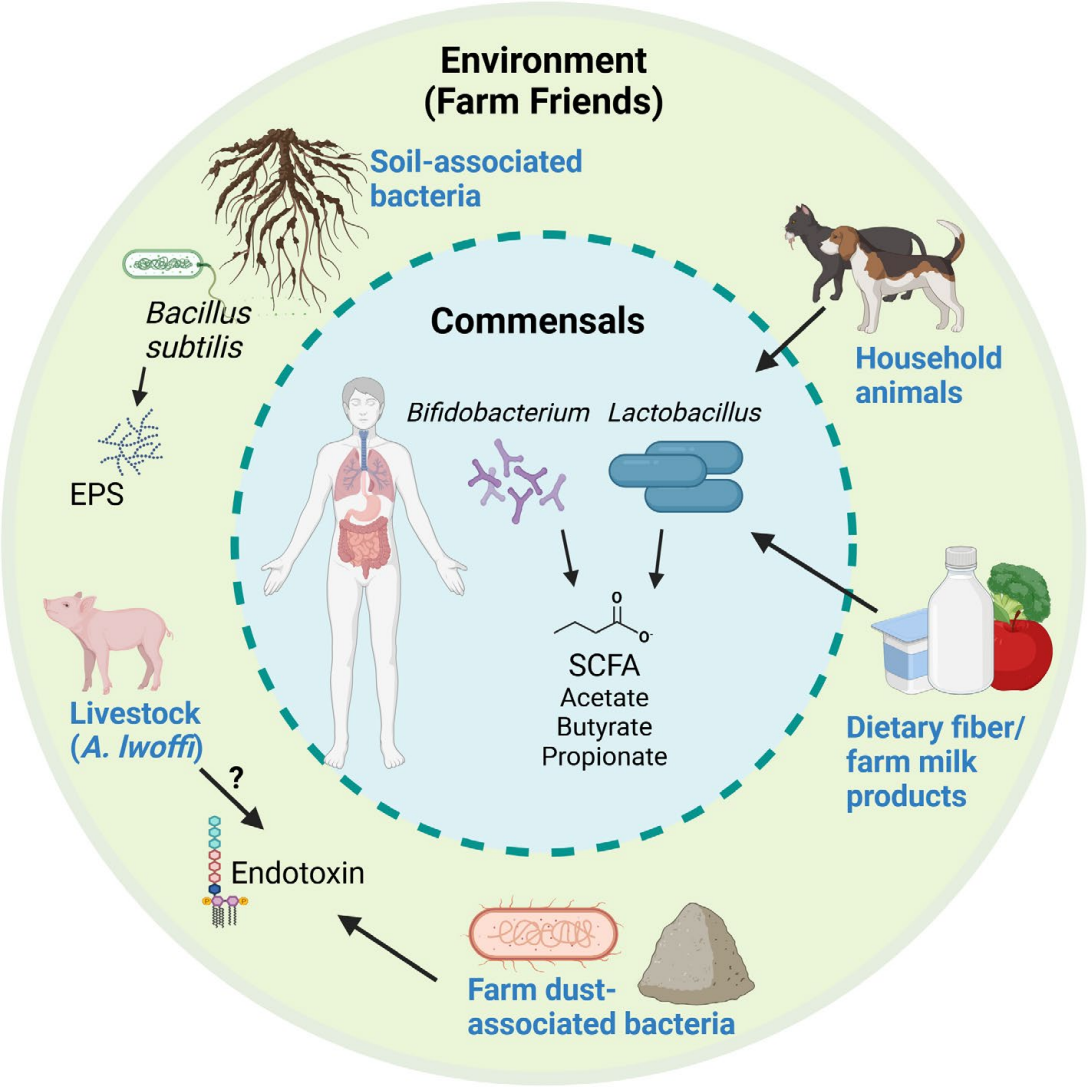
Farm‐Friends associated with protection from allergic asthma. Commensal bacteria belonging to the genus *Bifidobacterium* and *Lactobacillus* (inner circle) produce short‐chain fatty acids (SCFAs) such as acetate, butyrate, and propionate. In turn, these SCFAs maintain a tolerogenic environment in the gut and suppress pulmonary allergic inflammation through the gut‐lung axis. These commensals are also considered Farm‐Friends, as they can be introduced to the host by keeping cats or dogs (top right) or supplementation in unpasteurized milk (bottom right). Additionally, diets rich in vegetables and fiber provide the fuel necessary for these Farm‐Friends to make SCFA (bottom right). Other Farm‐Friends include soil‐associated microbes such as *B. subtilis* , which make EPSs that can suppress allergic inflammation (top left); *A. lwoffii* , a cowshed‐associated bacterium that promotes pulmonary type 1 immunity at the expense of pro‐allergic type 2 immunity (bottom left); and farm dust‐associated bacteria that produce the postbiotic product endotoxin. Figure generated in Biorender.

To better describe the intricacies of microbial exposure during this window and later in life, we are proposing new terminology, classifying these positive microbial identities as “Farm‐Friends”: microorganisms and their products linked to the rural lifestyle that aid in protection from asthma and allergy (Figure 1). These Farm‐Friends can come from various sources ranging from cow's milk to contact with domestic and farm animals, and to ingestion of less‐processed foods. Our understanding of how these Farm‐Friends influence the immune system has been growing over the years. In this review, we will address and discuss studies on how Farm‐Friends affect asthma risk and asthma severity. Once we understand the importance of these microorganisms, we will next explore how we can use them for preventive treatment. Finally, we will discuss the significance and relevance of localized probiotics based on the route of administration for the prevention and treatment of asthma throughout the lifespan.

## 2 Cellular Targets of Farm‐Friends in Allergic Asthma

To evaluate the specific interventions of Farm‐Friends in asthma disease, it is essential to first understand the mechanisms underlying asthma pathology. The allergic response requires a two‐phase immunological process consisting of an initial sensitization and challenge upon reencounter. Exposure to an allergen generates an innate response, where epithelial activation and/or damage of the airways results in the release of type 2 alarmins such as IL‐33, TSLP, and IL‐25. These cytokines activate type 2 innate lymphoid cells (ILC2s), eosinophils, and mast cells [24]. The allergen is taken up through the lung parenchyma by professional antigen‐presenting cells (APCs) such as dendritic cells (DCs), which then process the antigen and migrate to the lymph node. Here, these DCs can present allergen‐derived antigens and polarize naive T cells to differentiate into Th2 cells. These T cells migrate to the periphery and exert their adaptive effector functions in tandem with innate cells upon allergen reencounter [25].

Type 2 effector functions are driven by cytokine mediators including IL‐4, IL‐5, and IL‐13, which are produced primarily by ILC2 and Th2 cells. IL‐4 induces B cell class switching to IgE. Binding of antigen‐specific IgE to mast cells and basophils can result in downstream degranulation upon allergen binding to antigen‐specific IgE in immune complexes. IL‐5 is a major driver of eosinophil maturation, homing, survival, and activation. These eosinophils in turn serve to mediate inflammation via cytotoxic granule release. IL‐13 works directly on smooth muscle cells to contribute to airway hyperreactivity, as well as on epithelial cells to induce goblet cell hyperplasia and contribute to the characteristic mucus plug formation, a major cause of mortality in asthma [24].

## 3 Farm Effects: Natural Acquisition of Farm‐Friends and the Impact on Asthma Risk

In the last decade of the twentieth century, multiple groups observed that urban environments conferred a higher risk of atopic sensitization in children [26, 27] but the mechanism(s) by which urbanization increased atopic sensitization in children were not clear. A landmark study in Switzerland by Braun‐Fahrländer and colleagues found that, within a rural population, farmers' children had significantly lower rates of hay fever, current wheeze, and serum IgE against specific outdoor allergens than children of non‐farmers [28]. Shortly thereafter, a cross‐sectional survey of rural families in Austria, Germany, and Switzerland found that the prevalence of asthma was only 1% in children exposed to stables and farm milk during infancy, as opposed to 12% in children who were not exposed to either. Children with farm exposures were also less likely to have hay fever or atopic sensitization to common allergens [29]. From these findings and a subsequent multicenter European study that replicated these findings [30], Dr. Erika von Mutius developed the “Farm Effect” hypothesis, in which farm‐derived microbial exposures protect infants from the development of asthma and allergy at school age. These exposures are believed to result in “training” of the host immune system, such that the host remains hyporesponsive to environmental allergens upon re‐exposure [31].

What about the farm environment prevents asthma in exposed individuals? While many studies have demonstrated that farm exposure results in lower levels of atopic sensitization, hay fever, and wheeze [29, 32, 33, 34, 35, 36], others have found no association between childhood farm exposure and asthma prevalence specifically [37]. To clarify the role of the farm environment in protection from allergic asthma, Ege et al. stratified specific farm exposures and correlated individual exposures with asthma incidence in rural families. In the PARSIFAL study group, children exposed to pig farming, farm milk, animal sheds, or haying had a lower odds ratio of ever acquiring asthma. Conversely, children exposed to hares or rabbits had a higher odds ratio of acquiring asthma [38]. These findings were consistent with other studies in which exposure to non‐pasteurized farm milk or animal sheds [39, 40, 41] during pregnancy or infancy inversely correlated with asthma prevalence by age five. Consistent with this finding, exposures to farm dust have also been demonstrated to be protective against allergic sensitization in animal models [42, 43]. Even the presence of a “farm‐like” microbiota signature in non‐farming homes was found to be inversely correlated with asthma prevalence in children of those homes [44]. Together, these studies underline the importance of specific farm exposures and the microbiota associated with them in preventing allergic asthma throughout life.

## 4 Immunology of the Farm Effect: Studies of Amish and Hutterite School Children

**FIGURE 2:**
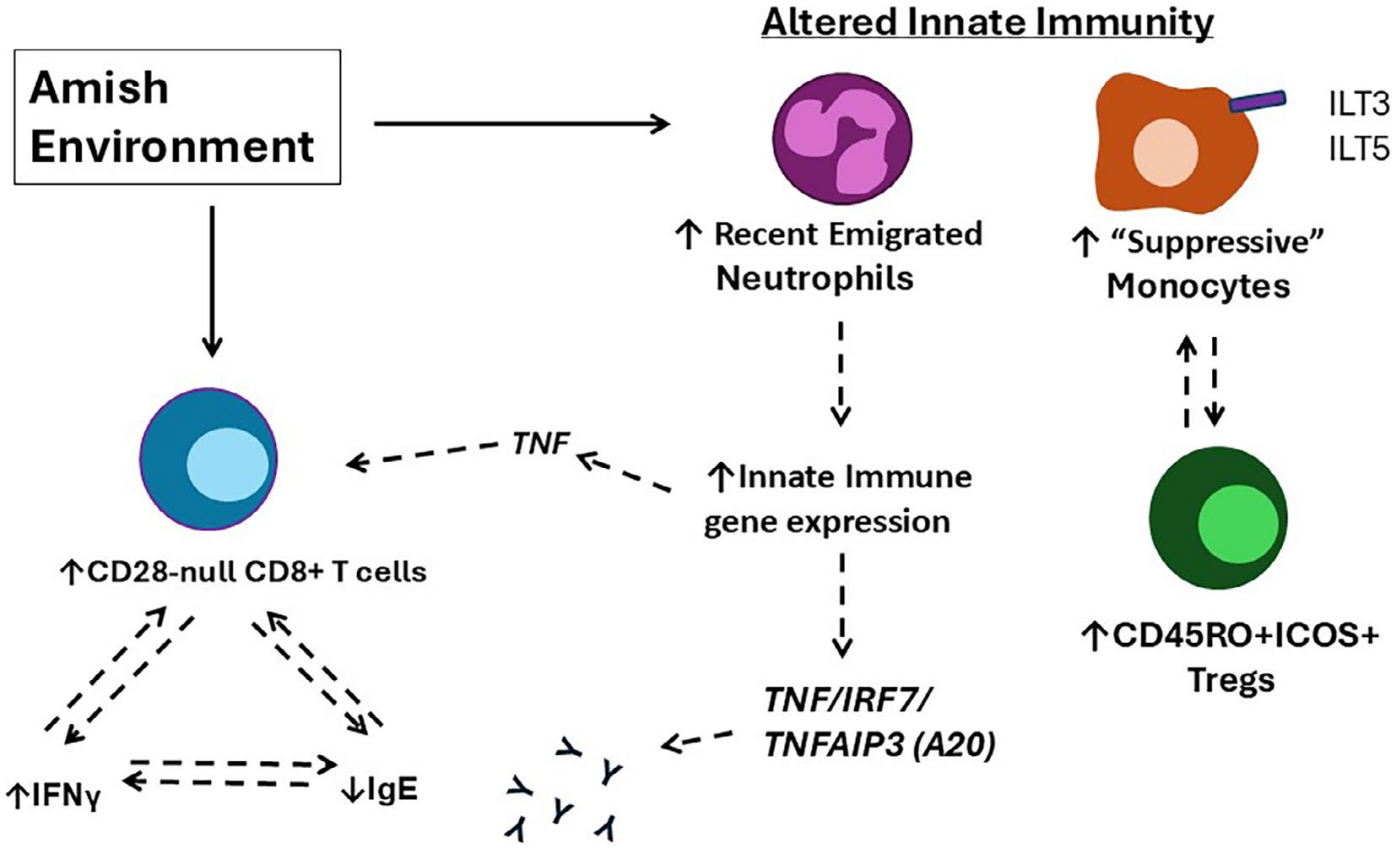
Effects of Amish environment on circulating immune cells: potential mechanisms for reduced asthma risk. Comparison of Amish and Hutterite circulating immune cells provided insights into the immune response that protects from asthma‐risk. In the Amish environment, innate cells that differed included neutrophils that were more abundant and had the phenotype of recently emigrated cells from the bone marrow. By RNA analysis, the neutrophils were found to have increased expression of innate inflammatory genes. Monocytes in Amish children expressed markers of a suppressive phenotype. The adaptive response in the Amish children's PBLs included increased activated Tregs, and increased CD28‐null CD8+ T cells that are characteristic of older adults and chronic viral infections. The dashed arrows represent significant correlations that were found. All data is from Stein et al. *NEJM*, 2016 [43] and Hrusch et al. *JACI* 2019 [45].

The effect of farm life on the immune system has been an active area of research for the past decades. In a cohort study of two agricultural communities with distinctly different rates of asthma and allergies, a new understanding of the Farm Effect evolved [43, 45]. With our colleagues, we have investigated the immunology of the US Amish and Hutterite farming communities, which had previously been shown to have distinct rates of asthma and allergies [46, 47]. Both groups are founder populations originally derived from nearby areas in Europe, yet the Hutterites have an asthma prevalence approximately 4× greater than the Amish. Due to the European ancestry of both groups, these communities are genetically similar to each other, which decreases the impact of genetic variation on allergic predisposition. While similar in many aspects, these two communities differ in their environmental exposures. The Amish operate small family farms close to home, use traditional non‐mechanic farming methods, and the entire family often contributes to farm chores. In contrast, the Hutterites live communally, operate industrialized farms that are frequently located away from the homes, and only men and older boys participate in farm work. By comparing Amish children's peripheral blood leukocytes (PBL) to cells from Hutterite children, we found that there were strong signs of constitutive innate activation [43] (Figure 2). In particular, neutrophils were increased, activated, and expressed markers that suggested that they were actively being recruited from the bone marrow, while monocytes had classic markers of over‐activation: decreased HLA‐DR expression and increased inhibitory receptor expression. Surprisingly, while the innate response at baseline was higher in the Amish children's PBL, after stimulation with LPS, the Hutterite PBLs produced higher amounts of many cytokines compared to the Amish children's PBLs. This hyperresponsiveness of the Hutterite children's PBLs to LPS was not limited to cytokines, as there was also a global increase in gene expression in the Hutterite children's PBLs compared to the Amish. Thus, while Amish children's immune system is at a higher baseline, they have a more measured response to innate stimulation than the Hutterite children's blood leukocytes.

As changes in the innate immune response affect the adaptive response, we investigated the adaptive response in the Amish and Hutterite children and the connections between their innate and adaptive responses [45]. Activated Tregs were increased in the Amish children's PBL, and surprisingly, the activated Tregs were directly correlated with the inhibitory receptor expression on the monocytes in the Amish, but not the Hutterite children. Interestingly, we also found that Amish children had a significantly higher percentage of CD28^null^ CD8 T cells, and these cells were correlated with increased IFNγ production and a reduction in circulating IgE in Amish, but not Hutterite children. The finding that healthy Amish children already had large numbers of CD28^null^ CD8 T cells was remarkable since these cells are generally associated with aging and chronic diseases, especially chronic viral infections [48, 49, 50, 51]. Together, these findings are in line with those predicted by the hygiene hypothesis: extensive exposure to a farm environment—similar to the Amish—can induce a higher baseline activation of both innate and adaptive immunity that protects against immune hyperresponsiveness. Understanding how the farm microbial environment induces an inhibitory response instead of an activating response remains one of the important unknowns of the Farm Effect.

## 5 Decreased Exposure to Farm‐Friends Alters the Lung and Gut Microbiota

The imbalance of pulmonary and gut microbial communities, resulting from urbanization and a correlating lack of microbial exposure, has been increasingly associated with asthma development and exacerbation [52]. This imbalance can involve the absence of specific microbial species that contribute to health, low diversity of the microbiota, or the presence of microbial species that are pathogenic. In asthma patients, the focus of the literature has widely involved the study of the gut microbiome and, more recently, the lung microbiome [53]. While the lung has been shown to have a bacterial density that is up to 10^2^ times less than the gut [54], the presence of a lung‐specific microbiome has contributed to the idea of a “gut‐lung axis”: the bidirectional crosstalk of microbial species in the lung and gut, as well as their metabolites, to facilitate and influence the overall health of each ecosystem. In a study describing the differences between healthy and asthmatic or COPD lung microbiota, *Bacteroidetes* such as *Prevotella spp*. were found to be more prevalent in healthy control subjects than in diseased lungs [54], a finding that also correlated with another study that demonstrated a 10‐fold decrease of *Bacteroidetes* in inflammatory bowel disease [55]. These data taken together indicate that microbial imbalances, whether they be the total absence or decreased prevalence of a bacterial phylum, can indicate and perhaps contribute to disease.

Crosstalk between the gut and lung microbiota has also been shown to contribute to the shaping and regulation of the lung immune response in mouse models of asthma. For example, dysbiosis of the gut induced by the fungus *Candida albicans* was found to correlate with increased eosinophilia in lung bronchial alveolar lavage (BAL) after house dust mite extract (HDM) exposure, but also that the ILC2 compartment is expanded in the lungs of dysbiotic mice even without HDM exposure [56]. Similarly, another study demonstrated that antibiotic depletion of the microbiome or the absence of a microbiome via germ‐free mice resulted in upregulation of the transcriptomic and epigenetic program of ILC3s in the gut, though there was an expansion of both ILC2 and ILC3 populations [57]. Another study in 2021 expounded on this idea of the gut microbiome influencing ILCs, and in fact determined that this effect can be systemic. The authors showed that Proteobacteria in the gut microbiome facilitates the increased production of IL‐33, which in turn drives increased accumulation of natural ILC2s in the lung [58]. This migration was found to be regulated by an IL‐33–CXCL16–CXCR6 axis; thus, the increase of Proteobacteria can indirectly influence ILC2 migration to and compartmentalization of the lung.

## 6 Harnessing the Farm Effect: Treating/Preventing Asthma Using Farm‐Friends Surrogates

Supplemental methods of administering beneficial microbes and their products have been developed to compensate for the absence of these Farm‐Friend exposures in daily life. Metchnikoff, who won the Nobel Prize in 1908, was one of the first to propose that the consumption of beneficial bacteria present in fermented milk could positively affect health. He observed that populations in Bulgaria, who regularly consumed yogurts rich in lactic bacteria such as Lactobacillus, had longer and healthier lives [59].

Fostering of a healthy microbiome in the absence of Farm‐Friends can be accomplished using multiple approaches. These approaches can be classified into three types of interventions including pre‐, pro‐, and post‐biotics. Prebiotics are fibers and nutrients that provide sustenance for beneficial commensals within the gut microbiome and are mainly found in the foods we eat [60]. These compounds are usually not digested by the human body, but by the bacteria that metabolize them. Probiotics are the live bacteria themselves that can be ingested in foods or manmade supplements [61]. Postbiotics are the metabolic products that beneficial microbial flora produce that are themselves beneficial to the health of the human host [62]. Through the administration of prebiotics, probiotics, and postbiotics, selective promotion of positive microbes can occur to improve overall human health and ameliorate allergic inflammation.

The use of probiotics to prevent or alleviate allergies has expanded. However, consensus on the effects of probiotics in asthma remains elusive due to complex interactions between environmental factors, microbiota composition, and an individual's genetic background. Given that approximately 4 million people in the United States consume probiotics and the market size is currently $12.5 billion (estimated to reach $39.5 billion by 2033), understanding probiotic effects on asthma is crucial [63].

## 7 Production of Short‐Chain Fatty Acids ( *SCFA* ) by Farm‐Friends: Promising Therapeutic Potential

Short‐chain fatty acids are the best‐known example of how intestinal probiotics can modulate respiratory immune function. SCFAs, after being produced in the intestine, enter the circulation and can reach different organs, such as the lungs, acting as one of the main connectors of the gut‐lung axis. Thus, the beneficial effects of these acids on asthma can occur both in the intestine where they are produced and in the lungs where they exert their effector function.

Natural and fiber‐rich diets, as often seen in rural lifestyles, favor the colonization of SCFA‐producing bacteria [64, 65]. SCFAs are formed through the fermentation of complex food fibers by certain bacteria [65, 66]. Acetate is the most abundant SCFA in the colon [66], and various bifidobacteria can produce it. Fermentation to produce another SCFA, butyrate, is carried out by other bacteria such as *Clostridium* ( *C. acetobutylicum* and *C. butyricum* ). Bacteria that produce the SCFA propionate include *Lactobacillus rhamnosus* GG, *Lactobacillus gasseri* PA 16/8, and *Akkermansia muciniphilia* [67]. The effects of these specific SCFAs in the prevention and treatment of various diseases, including allergies and asthma, are well documented [68, 69, 70, 71].

To choose a probiotic, one must understand how these microorganisms and their metabolic products interact with the host's immune system. In the case of allergic inflammation, SCFAs can be used to mediate the interaction between cells and the production of inflammatory cytokines. SCFAs can regulate type 2 inflammation by activating G‐protein‐coupled receptors (GPCRs; otherwise known as free fatty acid receptors [FFARs]), and inhibiting histone deacetylase (HDAC) activity. For instance, acetate is a potent agonist for GPR43, propionate is a potent agonist for GPR41, and butyrate is a potent agonist for GPR109A [72]. These metabolic sensors are expressed in various cell types, including immune cells such as neutrophils, eosinophils, DCs, ILCs, T cells, B cells, and macrophages, and non‐immune cells such as epithelial cells. While these sensors' functions have been extensively explored in the intestine [73], they are less studied in the lungs.

SCFAs can directly affect intestinal epithelial cells to protect against asthma exacerbations. The intestinal epithelium not only functions as an important barrier against harmful agents, but also participates in immune regulation [74, 75, 76]. When the integrity of the intestinal epithelial barrier is compromised, intestinal permeability to harmful substances increases, triggering local and distal inflammation [72]. It has been shown that there is an association between the breakdown of the intestinal barrier and asthma, but there is no consensus on whether this is a cause or consequence of the disease [77, 78, 79]. As SCFAs are fundamental for maintaining the integrity of the intestinal epithelial barrier [74, 75], their benefits for asthma may be due to direct effects in the intestine. Acetate and butyrate have been shown to be relevant for the restoration of the intestinal epithelial barrier through GPR43 and GPR109A receptors, respectively. Both promote the proliferation of epithelial cells after damage, restore tight junctions, and dampen the inflammatory response by increasing IL‐10 and inhibiting NF‐κB [80, 81, 82, 83, 84]. Emerging research suggests that SCFAs may also benefit respiratory epithelial health [85, 86]. In vitro and in vivo studies evaluating the effects of acetate, propionate, and butyrate on junction protein expression, epithelial barrier integrity, and inflammatory markers have identified that all three SCFAs, but especially acetate, have an important effect on maintaining respiratory epithelial homeostasis [87, 88]. Thus, the mucosal epithelium in both the gut and the lungs can be modulated by SCFAs and may contribute to their inhibitory effects on asthma.

During allergen exposure, epithelial cells are activated to release alarmins, which in turn activate ILC2s. Butyrate has been the most studied SCFA in the activation and proliferation of ILC2 in the context of asthma [89, 90], while propionate and acetate are less studied [90]. Recently, it has been shown that butyrate inhibits the activation of ILC2s and subsequently airway hyperreactivity [90]. By reducing ILC2 activation, the production of IL‐5 decreases, which can affect the migration of eosinophils to the airways, playing a crucial role in asthma [90, 91, 92, 93].

SCFAs can also interfere directly with eosinophils, as the receptors GPR43 and GPR41 are expressed on their surface. Although several studies have shown that SCFAs decrease eosinophil influx to the airways in experimental asthma [70, 94, 95], there are few studies on the mechanisms involved in this process. It has been shown that propionate and butyrate, but not acetate, play an important role in the apoptosis, endothelial adhesion, and migration of eosinophils, and these effects are mediated by HDACs [96]. Furthermore, neutrophils have high expression of GPR43, and in vitro studies demonstrate that acetate can affect their migration through actin polymerization and depolymerization [96, 97, 98]. Similar to eosinophils, propionate and butyrate can affect neutrophil apoptosis [96]. Although neutrophils are less relevant for type 2 asthma, approximately 1 in 5 adults with asthma have an increased proportion of neutrophils in the airways, usually occurring in older patients who do not respond to asthma medications [99]. Thus, SCFA administration becomes an interesting therapeutic strategy for other types of asthma.

DCs are relevant targets for SCFAs as they are fundamental to inducing a type 2 inflammatory response in the airways [100, 101]. In the presence of butyrate, in vitro studies have shown that they become less activated, with a reduction in co‐stimulatory molecules and reduced migratory ability [70]. Similarly to butyrate, propionate was also able to attenuate the ability of DCs to initiate a type 2 inflammatory response [94]. The effects of SCFAs on the adaptive immune response go beyond the DC‐T cell relationship. A significant mechanism of SCFAs in T cells involves inhibiting HDAC9 activity, leading to increased Tregs by derepressing FoxP3 and IL‐2 expression [95].

In addition to influencing regulatory T cells, acetate has also been shown to be important for regulatory B cells that produce IL‐10 (B10 cells). It has been demonstrated that acetate directly promotes the differentiation of B10 cells from B1a cells in mice, both in vivo and in vitro [102], and these B10 cells are important for controlling allergic pulmonary inflammation [103]. In humans, it has been shown that the consumption of complex fiber (a prebiotic) leads to an increase in B10 cells [102]. Furthermore, one study demonstrated that SCFAs influence B cell class switch recombination (CSR) to IgA both in vitro and in vivo [104]. Similar to IgA, SCFAs also inhibited CSR to IgE by acting directly on B cells to reduce both total and antigen (OVA)‐specific IgE titers. Intriguingly, experiments in germ‐free mice exhibit a significant reduction in IgA levels in the intestinal mucosa, highlighting the essential role of microbiota in IgA production [105]. Likewise, SCFA metabolites such as propionate and butyrate can affect antibody production as well [86, 106]. While the effects of SCFAs on different immune cell types are well documented, the therapeutic potential of dietary fiber and SCFAs in the context of allergic asthma specifically remains underexplored.

## 8 Exposure to Pets as a Source of Probiotics

Exposure to household pets such as dogs has been demonstrated to reduce child atopy and skin reactivity to allergens [107]. In 2014, Lynch and colleagues identified *Lactobacillus johnsonii* as a protective commensal species in dust from homes with dogs [108]. Using several mouse models of asthma, they demonstrated that dust from homes with dogs protected mice from type 2 responses in the lungs, but dust from homes without dogs had no effect. To identify microbiome involvement, they tested the cecum microbiome and found that a dominant *Lactobacillus* strain, *L. johnsonii*, was overrepresented in the protected mice. Oral supplementation of *L. johnsonii* was also sufficient to inhibit lung type 2 responses and airway hyperresponsiveness [108]. In RSV infection, *L. johnsonii* reduced lung IL‐4, IL‐5, and IL‐13, likely through the production of metabolites such as the fatty acid metabolite docosahexanoic acid [109]. Thus, metabolite production may be a mechanism through which *L. johnsonii* attenuates neutrophilic inflammation in the cockroach antigen (CRA) model. Oral supplementation with another Lactobacillus species, *Limosilactobacillus reuteri*, also alleviated HDM‐mediated allergic inflammation. *L. reuteri* ‐treated mice had a significant increase in cecal butyrate contents compared to sensitized controls, again highlighting the central role of SCFAs in modulating pulmonary allergic inflammation [110]. Both these *Lactobacilli* strains are used commercially in probiotic formulas and remain a target of experimental discovery [111, 112, 113].

In addition to dogs, cats can confer protection against asthma by modifying the owner's microbiota and consequently modulating the immune system [107, 108, 114]. A retrospective Japanese study showed that both dogs and cats protect their owners from the onset of asthma [115]. In contrast, a different study found that owning dogs and cats early in life does not decrease the risk of asthma in school‐age children but suggests that ownership may potentially exacerbate the risks associated with specific allergic sensitization to dogs and cats [116]. Another study showed that early‐life exposure to cats can attenuate the risk of developing asthma in genetically predisposed individuals, whereas the same results were not observed with dogs [117]. Therefore, the protection or increased risk that pets may offer for the development of asthma may be related to the genetic factors of the pet owner, as well as specific microbe(s) associated with feline exposure in childhood.

## 9 Agriculture‐Associated Probiotics: *Bacillus subtilis*

*Bacillus subtilis* , also known as the hay bacillus, has been associated with humanity for hundreds of years as the fermentation agent in the popular Japanese dish natto. Its use as a commercial probiotic in humans has also gained popularity in the United States and Europe [118]. Numerous studies have been conducted on the benefits of *B. subtilis* on host immunity in livestock. Feed supplementation with *B. subtilis* significantly increases intestinal villus height and crypt depth while also inducing expression of tight junction proteins ZO‐1, occludin, and claudin‐1 [119, 120]. Within the ileum, *B. subtilis* induced differentiation of secretory cells, including Paneth, goblet, and enteroendocrine cells, when administered orally over a 21‐day period. Furthermore, *B. subtilis* treatment increased expression of anti‐microbial peptides in the ileum, which resulted in resistance to *Salmonella typhimurium* infection [121]. Another important arm of protection from commensal and potential pathogenic bacteria is the production of luminal IgA in the gut. *B. subtilis* supplementation has been demonstrated to increase serum and luminal IgA after supplemental feeding in broiler chicks [119, 122] and weanling pigs [111]. Increases in serum IgA after *B. subtilis* feeding were also associated with reduced severity of diarrhea in beagles challenged with enterotoxigenic *E. coli* [123].

In light of these findings, the use of *B. subtilis* as an oral probiotic to suppress allergic inflammation has gained recent traction. Specifically, oral gavage with *B. subtilis* prior to sensitization and challenge with HDM significantly suppressed eosinophils in the BAL fluid [124]. This protection extended up to 4 weeks post‐gavage and corresponded with an increase in BAL macrophages, a cell subset present in the BAL at homeostasis. *B. subtilis* gavage also corresponded with alterations to the fecal microbiome, indicating that the anti‐allergic effects of *B. subtilis* may extend to its interaction with other commensals [125].

What active component(s) of *B. subtilis* are responsible for this anti‐allergic effect? Swartzendruber and colleagues demonstrated that oral gavage of *B. subtilis* spores lacking the ability to produce exopolysaccharide (EPS) was not as protective against allergic inflammation as EPS‐sufficient spores [124, 125]. A second, independent group demonstrated that EPS gavage from a different strain of *B. subtilis* increased the concentration of antioxidants in the BAL and suppressed total transcription of the pro‐inflammatory mediators NF‐κB, JAK1, and STAT6 [126]. Neither study, however, isolated the cell type(s) engaged by EPS to exert its protective effects. EPSs produced by different microbes are readily found in house dust; therefore, exposure to EPS may be implicated in protection from allergic asthma. Consistent with this notion, children from farm homes with EPS‐rich dust had lower odds of being asthmatic in a 2007 study, even after adjusting for sex and family history of asthma [38]. Thus, the possibility of harnessing EPS as an anti‐asthma prophylactic has potential, but much work remains to be done regarding feasibility and mechanism.

## 10 Dietary Pre‐ and Probiotics of the Gut: Dairy Products

One of the central exposures associated with the Farm Effect is maternal or neonatal exposure to cow's milk [29, 41, 127, 128]. However, cow's milk is one of the most common allergens in children [129, 130, 131, 132]. Studies addressing the effects of milk on asthma show varied results, which may be due to several differences among them, such as whether pasteurized or unpasteurized milk was studied [128, 129, 133]. While pasteurization removes potential pathogens and extends milk's shelf life [134], it may also remove probiotics capable of “reprogramming” the host immune system against the development of asthma and allergies. Because of the health risks associated with unpasteurized milk, isolation of the individual component(s) in milk that prevent allergic sensitization is of paramount importance.

Breast milk, both human and bovine, is a rich source of oligosaccharides, immunoglobulins, and vitamins meant to nourish offspring and impart immune protection until weaning. Upon reaching the host intestine, milk sugars such as lactose can be metabolized into SCFAs [135]. Furthermore, raw milk itself induces changes in the host microbiota by increasing putative butyrate‐producing bacteria [136] and introducing immunomodulatory bacteria such as *B. subtilis* that prevent the outgrowth of pathogens [137]. In one study, heat‐labile products in bovine milk were demonstrated to induce CD103^+^CD11b^+^ DCs and Tregs in the mesenteric lymph nodes [138]. Raw cow's milk also contains the immunomodulatory cytokine TGF‐β [135], which can induce antigen‐specific tolerance in the offspring of airborne antigen‐exposed mothers [139]. Other bovine‐derived proteins, such as the lipocalins OBP and Bos d 2, have been identified in farm‐derived dust extracts that protect against chicken ovalbumin (OVA)‐induced allergic inflammation in murine models [140]. Thus, there are a plethora of potential immune mediators in raw milk that could be explored through prophylactic or therapeutic avenues.

Another dairy‐related source of probiotics and postbiotics is fermented milk. Fermented foods are generally defined as foods and beverages created through desired microbial growth and enzymatic conversion of food components [141]. Fermented dairy products such as yogurt, kefir, and cheese are key sources of living microorganisms, mainly lactic acid bacteria (LAB), that are capable of modifying the immune response [142, 143, 144, 145]. An example of a probiotic‐rich fermented dairy beverage is kefir, a product similar to yogurt, whose main metabolic product is lactic acid [146]. LAB strains derived from kefir, such as *L. kefiranofaciens* and *L. kefiri*, have also shown anti‐allergic effects by increasing IFNγ and dampening the type 2 response [145, 147]. Kefir has been found to regulate type 2 responses due to high levels of *L. kefiranofaciens* M1 isolated from kefir grains. *L. kefiranofaciens* reduces IL‐5 levels, increases the percentage of Tregs, and inhibits IgE production in OVA‐sensitized Th2‐polarized mice [147]. Another study also reinforces the ability of kefir to increase Tregs, regulate the allergic response, and reduce mucus in the airways and bronchial hyperreactivity in OVA‐sensitized mice [148]. In general, fermented dairy products have been shown to be beneficial by decreasing the expression of FcεRI genes and suppressing mast cell degranulation [149, 150]. There are also studies implicating increased eosinophil apoptosis and reduced eosinophil infiltration in the airways due to lower adhesion to endothelial cells [148, 151, 152]. Dr. Ferreira and colleagues have evaluated the effects of kefir administration on the exacerbation of allergic disease in ovariectomized animals, a model of post‐menopausal asthma [153]. Kefir was administered daily for 10 days before the first sensitization with OVA, and its administration continued until 24 h before ovary removal. This treatment was able to reduce cellularity in bronchoalveolar lavage, especially eosinophil influx, reduce IL‐13, abrogate mucus production in the bronchi, and modulate macrophage identity by reducing M2 macrophages [153]. Other studies show that kefir is also relevant for bone metabolism, preventing osteoporosis in menopause, indicating that dairy products can provide benefits other than immunomodulation for menopausal women.

## 11 Probiotics of the Gut: *Bifidobacterium longum 5* ^*1A*^

**FIGURE 3:**
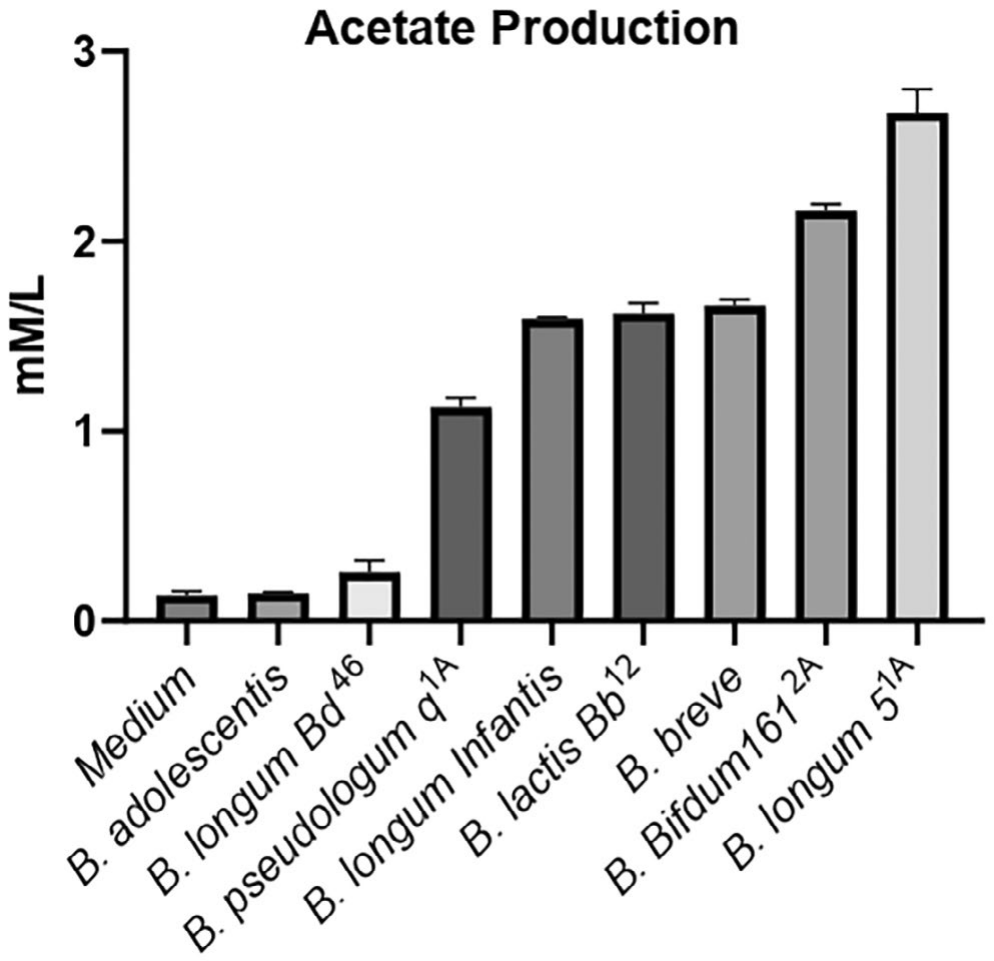
Optimizing probiotic strains: *B. longum* 5^1A^ leads in acetate production. Acetate production varies among *Bifidobacterium* (B) strains. All bifidobacteria were cultured in MRS medium for 48 h then analyzed for acetate concentration using gas chromatography. *B. longum* 5^1A^ exhibited the highest acetate production compared to other bacteria, while *Bifidobacterium adolescentis* produced the least acetate [154, 155]. This tool is an example for identifying optimal SCFA‐producing probiotic strains that effectively modulate the immune system.

Research from Dr. Ferreira's group has evaluated the effects of probiotics in a mouse model of experimental allergic asthma. We assessed the production of the SCFA, acetate, in cultures of various bifidobacteria. *Bifidobacterium longum* 5^1A^ was selected due to its ability to produce acetate in greater quantities as compared to other species (Figure 3). Given the known anti‐inflammatory effects of acetate in various conditions [156, 157], including asthma, *B. longum* 5^1A^ could act more effectively. This bacterium was originally isolated from the intestine of a healthy 5‐year‐old child and was identified by Gram staining, growth atmosphere, and biochemical tests, followed by multiplex PCR [158]. Our initial studies aimed to evaluate whether *B. longum* 5^1A^ could protect from the development of asthma. We found that the administration of *B. longum* 5^1A^ orally for two weeks before sensitization and challenge with OVA resulted in changes in gut microbiota [159]. Surprisingly, we found varying effects on allergic lung inflammation; A/J mice exhibited reduced eosinophilia and other allergic parameters, while C57BL/6 mice showed increased eosinophilia [159]. Notably, A/J mice had increased serum acetate levels 15 days after probiotic treatment, whereas C57BL/6 mice had decreased acetate levels [159]. To demonstrate that differing levels of acetate were responsible for these effects on lung inflammation, we treated the A/J mice with acetate and replicated the reduced eosinophil response seen in *B. longum* 5^1A^‐treated mice [159]. These data suggest that the microbiome interaction with host genetics can impact the production of SCFA and change the result of probiotic treatments. Indeed, low acetate levels in children correlate with an increased risk of developing asthma during school age [160]. These findings highlight the importance of probiotic selection and their interaction with both genetics and the immune system.

## 12 Probiotic in the Gut: For Later Life

Although there are systematic reviews providing information on the use of probiotics for the treatment of diarrhea and dementia [161, 162], the benefits of probiotics, prebiotics, and postbiotics for the elderly still need to be better investigated, especially for preventing and treating asthma and its exacerbations. The changes in the microbiota that occur during mid and later life can be different between men and women, mainly due to menopause and its hormonal changes [163].

It is important to note that the human gut microbiome changes throughout life, occurring mainly after weaning [164, 165]. Besides the weaning phase, another phase of life where changes in the gut microbiota are also important is around puberty, a period when the male and female microbiota differentiate. A study showed that pairs of adolescent dizygotic male and female twins have greater microbiome dissimilarity than pairs of same‐gender dizygotic twins, a pattern not observed in childhood [166, 167]. Studies in mice show the differences between the microbiota of males and females after puberty and discuss how these differences correlate with the higher incidence of autoimmune diseases in women [168, 169]. Interestingly, asthma also shows sexual dimorphism during puberty. Early‐life asthma is more prevalent in boys, but this reverses at puberty, becoming more prevalent in girls, and several studies report that disease symptoms can be exacerbated in the premenstrual phase when female sex hormones (FSH) are lower [170]. Thus, it is possible that the hormonal fluctuations that occur in the premenstrual phase alter the microbiota, exacerbating airway inflammation and worsening asthma symptoms.

These sexual differences in gut microbiota remain little explored during the aging process, especially concerning menopause, a milestone in women's reproductive aging. Interestingly, just as during the menstrual cycle where there are drops in estrogen levels, in perimenopause, FSH levels begin to decrease, but at the same time, their levels fluctuate, exacerbating asthma [171]. Although sex hormone levels affect the microbiota, the microbiota can also regulate the levels of free circulating hormones, suggesting a bidirectional interaction [172]. Endogenous estrogens are metabolized in the liver through irreversible first‐pass hydroxylation and/or conjugation with glucuronide or sulfate groups, allowing the biliary excretion of estrogens into the intestine, where some gut microbiota bacteria can deconjugate estrogens, thus being reabsorbed and reaching circulation to target cells in tissues [173]. The genes of gut microbiota bacteria capable of performing this deconjugation are called the estrobolome [174, 175]. These bacteria are mainly from the Firmicutes and Bacteroides phyla, bacteria that possess beta‐glucuronidases. Besides these important aspects of the microbiota in menopause, it is also reported in the literature that in menopause there is a reduction of SCFA‐producing bacteria [176, 177, 178]. As mentioned above, SCFAs are related to anti‐inflammatory effects.

Considering all these aspects of worsening asthma symptoms in perimenopause, alteration of gut microbiota, and decrease in SCFAs, we investigated the effect of *B. longum* 5^1A^ in a postmenopausal mouse model of asthma. The action of probiotics in preventing these symptom exacerbations that occur in menopause has been little explored. To better investigate the mechanisms and role of the microbiota in the exacerbation of asthma during menopause, we developed an animal model where the animals are sensitized and challenged with an allergen, then ovariectomized and re‐challenged with the allergen again. In this model, there is a strong exacerbation of type 2 inflammation compared to sham‐operated animals (sham group) [154]. The model mimics what happens in some asthmatic women when they enter menopause. Treatment with the acetate‐producing probiotic led to a reduction in the exacerbation of T2 airway inflammation and an increase in Treg cells in the bronchoalveolar lavage [154]. We also observed that the ingestion of *B. longum* 5^1A^ was positively correlated with acetate levels in the feces of re‐challenged and ovariectomized allergic animals [154]. Thus, probiotics may provide a novel treatment for the increased asthma severity suffered by many postmenopausal asthmatics.

## 13 Location, Location, Location: Potential for Inhaled Pro‐ and Post‐Biotics in the Airways

In recent years, the importance of tissue‐specific immunity has gained traction in the field of vaccinology. For example, preclinical vaccination studies have found that intranasal vaccination boosts antiviral CD8^+^ T cells, IgG, and IgA in the respiratory tract compared to intramuscular vaccination [179]. Therefore, there has been a burgeoning interest in modulating respiratory inflammation through the administration of intranasal probiotics and postbiotics. The use of nasal probiotics is quite recent, with the first research emerging in the early 2000s [180]. The administration of probiotics and their derivatives for the treatment of non‐infectious chronic diseases has been less studied, as most studies in this area have focused on immune responses to infection [181]. In addition to live bacteria, products from their cell walls and their metabolites have also been used to prevent or treat allergies. Here, we discuss respiratory probiotics and postbiotics—many informed by discoveries of the Farm Effect—that have displayed promise in suppressing allergic inflammation in murine models.

## 14 Inhaled Probiotics: *OM* ‐85

The bacterial lysate OM‐85 was first developed to prevent respiratory infection; however, oral administration of OM‐85 in rats and mice suppressed the development of allergic inflammation in the lungs [182, 183]. It was hypothesized that OM‐85's anti‐allergic activity would be as, if not more, efficient if given in the respiratory tract. Indeed, OM‐85 exposure through the intranasal route was able to inhibit eosinophilia and improve pulmonary function in two different murine models of experimental asthma: OVA/alum and *Alternaria alternata* [184]. In both models, OM‐85 induced FoxP3+ Tregs, downstream of an increase in CD103 + CD207+ cDCs in the lung. The use of intranasal OM‐85 prior to *A. alternata* sensitization reduced BAL IL‐33 levels and lung ILC2s, both factors crucial for initiating allergic inflammation in mice and humans. Interestingly, this protection was microbiota‐independent, as germ‐free mice were also protected against *A. alternata* ‐induced allergic inflammation after intranasal administration of OM‐85 [185]. Further study is required to determine which active microbial product(s) within the OM‐85 lysate are necessary and/or sufficient to prevent IL‐33 release, ILC2 accumulation, and accumulation of Tregs within the lung tissue.

## 15 Inhaled Probiotics: *Acinetobacter lwoffii*

Multiple groups have attempted to isolate farm shed‐associated microbes that have immunomodulatory properties in vivo. One gram‐negative bacterium, *Acinetobacter lwoffii*, stood out due to its relative abundance in cowshed environments, an exposure inversely associated with asthma prevalence [38]. A cowshed isolate of *A. lwoffii*, when administered intranasally to adult mice prior to sensitization with OVA/alum, prevented eosinophilia in the BAL and improved airway reactivity [39]. Consistent with the idea that prenatal exposures are also sufficient to protect against allergic inflammation, the pups of dams treated with *A. lwoffii* were protected against lung eosinophilia, goblet cell metaplasia, and overall heightened lung inflammation when challenged with OVA 6 weeks after birth [186].

When administered intranasally over multiple weeks, *A. lwoffii* increased levels of the pro‐Th1 cytokines IL‐6, TNF‐ɑ, and IL‐1β both systemically and in the BAL fluid. These mice were protected from pulmonary inflammation induced by sensitization to OVA in an IL‐6‐dependent manner [187]. Notably, *A. lwoffii* produces endotoxin, another farm exposure inversely associated with the occurrence of hay fever, atopic asthma, and atopic sensitization [188], which may contribute to its inhibitory properties.

## 16 Inhaled Postbiotics: Endotoxin

With a smaller microbial burden than the intestine, the lung may be exquisitely sensitive to exogenous probiotics, especially if the probiotic in question is capable of outgrowth to the point of infection. For example, the bacterium *A. lwoffii* can act as an opportunistic pathogen, especially in immunocompromised children [189]. Therefore, another potential strategy to prevent allergic asthma is the administration of postbiotics to modulate host immunity without outgrowth of the source probiotic.

Perhaps one of the oldest known postbiotics is endotoxin. LPS, a type of endotoxin, is a bacterial product found within the cell wall of Gram‐negative bacteria and is primarily sensed by the pattern recognition receptor TLR4. TLR4 engagement leads to activation of the master transcription factor NF‐κB and is, therefore, crucial for the activation of innate and adaptive immunity [190]. Repeated doses of endotoxin, however, can render the target cell hyporesponsive to TLR4 stimulation, a phenomenon known as “endotoxin tolerance” [191]. In vitro, both mouse and human myeloid cells secrete fewer pro‐inflammatory cytokines such as TNFα, IL‐6, IL‐12, and IL‐1β upon exposure to a second dose of endotoxin. Endotoxin‐tolerized myeloid cells instead secrete anti‐inflammatory cytokines such as IL‐10 and TGF‐β [192].

Concurrent with the development of the Farm Effect hypothesis, Dr. Erika von Mutius and colleagues found that indoor endotoxin levels were significantly higher in the dust from farm homes than from the homes of rural, non‐farming families [43, 188, 193, 194, 195]. Endotoxin levels in household dust and mattress dust, especially in farming families, were inversely correlated with the occurrence of atopic sensitization, hay fever, and allergic asthma in children, leading multiple groups to hypothesize that endotoxin tolerance was associated with the Farm Effect. The anti‐allergic effect of environmental endotoxin, however, remains controversial; at least one group has found a non‐significant association between endotoxin exposure and the prevalence of allergic asthma [38], and endotoxin exposure in house dust has also been positively associated with asthma symptom severity in adults [196, 197, 198].

While endotoxin pre‐exposure can protect against eosinophilia and other manifestations of type 2 inflammation, even a slight change in the dosage and/or timing of endotoxin exposure could exacerbate inflammation. In low doses, pulmonary endotoxin co‐administered with OVA **induces** Th2 responses upon subsequent challenges with OVA in mice [199, 200, 201], while pre‐exposure to endotoxin at the same doses protects against allergic inflammation [201]. In the context of HDM‐induced allergic inflammation, chronic low‐dose endotoxin exposure two weeks prior to sensitization prevented eosinophilia, IgE production, and airway hyperresponsiveness, phenocopying the epidemiological association between endotoxin exposure and lower odds of asthma diagnosis [188]. This anti‐allergic effect was mediated through upregulation of A20, a negative regulator of TLR4 signaling, in epithelial cells [202]. In multiple allergy models, treatment with larger doses of endotoxin results in suppressed eosinophilia and mucus secretion in airways, but also enhances neutrophilia and disrupts alveolar architecture [199, 203]. Therefore, the application of endotoxin as a respiratory postbiotic carries a significant risk of a deleterious inflammatory response.

## 17 Inhaled Postbiotics: Exopolysaccharide ( *EPS* )

EPSs are a class of high molecular‐weight polysaccharides secreted by microbes within a biofilm. EPSs vary in structure depending on their microbe of origin, and a single microbe can secrete multiple EPSs [204]. However, the composition of EPSs within a single species remains remarkably consistent across carbon sources [205]. Like endotoxin, EPS levels in house dust have been inversely correlated with asthma diagnosis in children since the Farm Effect was first described [195, 206, 207]. The hay bacillus *B. subtilis* produces large amounts of EPS in soil‐associated biofilms [208] and is readily isolatable from dairy cows [137], a component of farm exposure that is inversely correlated with asthma diagnosis and atopy [38, 39].

The presence of *B. subtilis* and EPS in the farm environment led us to hypothesize that EPS from *B. subtilis* may be another Farm‐Friend‐derived postbiotic that can protect against allergic sensitization. Therefore, we investigated the ability of EPS isolated from *B. subtilis* to prevent allergic inflammation when given intratracheally. Indeed, respiratory pre‐exposure to EPS prevented eosinophilia, goblet cell metaplasia, and Th2 accumulation in a HDM model of allergic asthma. Loss of TLR4 in myeloid cells abrogated protection from allergic sensitization, leading us to hypothesize that EPS prevents DCs from successfully priming T cells. Consistent with this hypothesis, DC pre‐exposure to EPS prevented successful internalization of HDM allergen and downregulated transcripts of key innate cytokines, such as TNF‐α (Hollinger, et al. Unpublished data). Excitingly, intratracheal distillation with EPS alone did not change the immunological environment of the lung, despite being given at a fourfold higher dose than the dose of endotoxin needed to induce significant lung neutrophilia [203]. These studies highlight the exciting potential of *B. subtilis* EPS as a prophylactic against allergic asthma.

## 18 Harnessing the Hygiene Hypothesis: Where Do We Go Next?

The decades of investigation into the Farm Effect and the causative agents therein have opened up a rich field of possibilities for preventing and treating allergic asthma. The Farm‐Friends described here, including *Lactobacillus* spp., *A. lwoffii* , *B. longum* 5^1A^, and *B. subtilis* , each modulate host immunity in unique ways. Specifically, gut‐associated *Lactobacillus* and lactic‐acid bacteria produce short‐chain fatty acids with a range of anti‐allergic effects through the gut‐lung axis. Inhaled *A. lwoffii* inhibited type 2 responses in the lung in favor of Type 1 immunity, thereby biasing lung immunity against inflammation against inhaled allergens. *B. subtilis* can modulate allergic inflammation through altering gut microbiota and production of EPS, which can suppress allergic inflammation when given orally or through the respiratory tract. *B. longum* 5^1A^ protects from airway inflammation both in adult mice and induced menopause mice through the production of acetate. Isolating the specific molecule(s) produced by these Farm‐Friends enables not only a mechanistic understanding of how allergic inflammation is initiated and prevented but also provides a potential intervention strategy for preventing allergic sensitization. Furthermore, acetate‐producing probiotics can modulate allergic inflammation later in life, as demonstrated by our own work in the ovariectomized models of post‐menopausal asthma. There is a significant gap in understanding how probiotics and prebiotics interact with medications, such as hormonal replacement therapy in menopause, and their health consequences, especially for exacerbations and triggering of asthma. Furthermore, if farm food products rich in phytoestrogenic compounds can act as a substitute for artificial hormonal replacement therapy, and the importance of these phytoestrogenic compounds in restoring the microbiota post‐menopause, is an important question in the field. Further investigation of other Farm‐Friends products in not only preventing but also treating pre‐existing allergic inflammation remains a wide‐open field with exciting implications.

## Conflicts of Interest

The authors declare no conflicts of interest.

## Data Availability

The authors have nothing to report.
